# Malformation of Alimentary Canal*A paper read before the Veterinary Medical Society in connection with the Ontario Veterinary College.

**Published:** 1893-02

**Authors:** A. G. Bowker


### MALFORMATION OF ALIMENTARY CANAL.*

By Mr. A. G. Bowker.

During the past Summer we were called to the country to see the following case: On arrival we found our patient to be a filly foal one and a half days old, and as yet had had no passage from the bowels.

*Symptoms* as follows:—Unable to stand without support, rolling about, straining at times, pulse 122, full and bounding, temperature $101\frac{2}{5}$, extremities cold.

*Diagnosis.*—Constipation.

*Treatment.*—Gave internally Linseed Oil, one ounce, Castor Oil, one ounce, Whiskey, two drachms, in water and mother's milk. Gave copious injections of warm water and soap. Drew off the urine, hand rubbed the abdomen, and applied a mild liniment and wrapped a blanket around body. We remained four hours, at the end of that time our patient seemed a little easier, but the bowels remained inactive. Within thirty hours we were again called to attend her, and found her weaker than before. Pulse 142, temperature 102 degrees, suffering considerable pain, and as yet had passed no fæces. Gave a drench composed of Castor Oil, one ounce, Flemings Tr., two minims, Syrup Buckthorn, one drachm, Tinc. Opii, half drachm, in warm water and milk. Followed with enemas and hot cloths to abdomen. I inserted catheter into anus, but met with no resistance. Death closed the scene two hours later.

*Post-Mortem.*—Found mucous coat of small intestines nearly black. Great colon was somewhat distended and contained hard pellets of meconium and had no communication with the floating colon which commenced from a blind extremity and was connected to the great colon by fibrous and adipose tissue only. Floating colon and rectum were empty. Abdominal cavity contained considerable fluid of a yellowish color. Stomach nearly full of fluids.

---

* A paper read before the Veterinary Medical Society in connection with the Ontario Veterinary College.

